# Effects of Electroacupuncture on Gastrointestinal Motility Function, Pain, and Inflammation *via* Transient Receptor Potential Vanilloid 1 in a Rat Model after Colonic Anastomoses

**DOI:** 10.1155/2022/5113473

**Published:** 2022-07-05

**Authors:** Xuelai Zhong, Zhaodi Zhang, Jiaying Li, Dandan Liu, Chao Ma, Guonian Wang

**Affiliations:** ^1^Department of Anesthesiology, Harbin Medical University Cancer Hospital, Harbin 150081, China; ^2^Department of Anesthesiology, The Fourth Hospital of Harbin Medical University, Harbin 150081, China; ^3^Department of Anesthesiology, The Fourth Hospital of Harbin Medical University; Pain Research Institute of Heilongjiang Academy of Medical Science, 150 Haping Rd, Nangang District, Harbin 150081, China

## Abstract

**Background:**

Complications after colon surgery are a major obstacle to postoperative recovery. The purpose of this study was to investigate the effect of electroacupuncture (EA) at Zusanli (ST36) on gastrointestinal motility in rats after colonic anastomosis and the mechanism of transient receptor potential vanillin 1 (TRPV1) channel in regulating gastrointestinal motility, pain, and inflammation.

**Methods:**

The rats were randomly divided into six groups, including the control, model, EA, sham-EA, capsaicin, and capsaicin+EA groups, with preoperative capsaicin pretreatment and EA treatment at ST36 acupoint after surgery. Rats were treated using EA at ST36 or sham acupoints after surgery for 5 days. Capsaicin was intraperitoneally injected into rats 3 hours before surgery. Gastrointestinal motility was assessed by measuring the gastric residue, small intestinal propulsion *in vivo*, contractile tension, and frequency of isolated muscle strips *in vitro*. The mechanical withdrawal threshold (MWT) of abdominal incision skin and spontaneous nociceptive scores were observed and recorded in rats after colon anastomosis. The expressions of TRPV1, substance P (SP), neurokinin 1 (NK1) receptor, nuclear factor kappa-B (NF-*κ*B), interleukin- (IL-) 6, L-1*β*, and tumor necrosis factor- (TNF-) *α* were determined.

**Results:**

Compared with the model group, electroacupuncture at ST36 point could significantly reduce the residual rate of stomach in rats after operation and increase the propulsive force of the small intestine and the contraction tension of the isolated smooth muscle. Electroacupuncture also increased postoperative day 3 MWT values and decreased postoperative spontaneous nociception scores. In addition, electroacupuncture treatment downregulated the expressions of IL-6, IL-1*β*, TNF-*α*, TRPV1, NF-*κ*B, SP, and NK1 receptors in the colon tissue of rats after colonic anastomosis.

**Conclusions:**

Our study showed that electroacupuncture at ST36 acupoint could improve gastrointestinal motility in rats after colonic anastomosis and relieve intestinal inflammation and pain. The mechanism may be to inhibit the activation of NF-*κ*B and SP/NK1 receptor signaling pathways by inhibiting TRPV1.

## 1. Introduction

Electroacupuncture (EA) is a modified technique that provides a small electrical stimulus to achieve better therapeutic outcomes, which is considered a safer, more effective, and less toxic therapy compared with conventional acupuncture treatment [1]. It has been demonstrated that the Zusanli (ST36) acupoint is frequently and effectively used for gastrointestinal diseases [2]. EA stimulation is in widespread use to treat different types of gastrointestinal diseases including disordered jejunal motility induced by stress [3] and functional dyspepsia [4]. Despite these previous studies, there are few studies on the effects of EA on the gastrointestinal motility function in rats after colonic anastomosis and on postoperative-related responses.

Transient receptor potential Vanilloid 1 (TRPV1) is a member of the transient receptor potential family and is expressed in the gastrointestinal tract, can be activated by various tissue injuries and validation of endogenous stimuli generated, and is also a key modulator of visceral hyperalgesia [5, 6]. Animal researches have investigated the role of TRPV1 in alleviating visceral hyperalgesia and attenuating colon transit in the irritable bowel syndrome animal models [7]. It has been suggested that TRPV1 appears to play an important role in the inhibition of jejunal motility in an intensity-dependent manner via the sympathetic pathway in EA. [8] A previous study showed that EA could significantly downregulate the increased TRPV1 expression from peripheral dorsal root ganglion to central spinal cord to reduce chronic inflammatory pain [9]. However, the mechanism of action of TRPV1 in EA on gastrointestinal motility function and postoperative inflammatory pain after colonic anastomosis in rats is unclear.

Nuclear factor kappa-B (NF-*κ*B) is an important nuclear transcription factor in cells involved in inflammation, immunity, pain, and stress responses [10]. NF-*κ*B signaling and gene expression of its cytokines have been associated with acupuncture points [11]. In addition, it has reported that the analgesic effects of acupuncture can be attenuated by reducing IL-6 levels and the proportion of CD68 macrophages *via* using NF-*κ*B inhibitors [12]. However, whether the effects of EA on postoperative gastrointestinal motility function, postoperative pain, and inflammation are mediated *via* the TRPV1 pathway, and NF-*κ*B transcription is not unequivocal.

In this study, we utilized a rat model after colonic anastomosis to investigate the effect of ST36 acupuncture on gastrointestinal motility, postoperative inflammatory response, and pain and further explored the potential mechanism of TRPV1 and NF-*κ*B in electroacupuncture at ST36 point after colonic anastomosis in rats.

## 2. Methods

### 2.1. Animals

Female Wistar rats (8-10 weeks old, 250 to 300 g) were obtained from Vital River Laboratory (Beijing, China). All rats were housed in an animal room (22 ± 2°C, 12 h light-dark cycle). Rats had unlimited access to water and food. Rats were acclimated to the environment for 5 days before the experiment. All animals adhered to the Guidelines for the Care and Use of Laboratory Animals by the National Institutes of Health. The protocol of current study was approved by the Medical Ethics Committees of Harbin Medical University Cancer Hospital.

### 2.2. Experimental Design

The rats were randomly divided into six groups (8 rats in each group), including the control group, not treated; the model group, colonic anastomosis; the EA group, EA treatment at ST36 acupoint after surgery for 5 days; the sham-EA group, EA treatment at sham acupoint after surgery for 5 days; the capsaicin group, preoperative capsaicin pretreatment; and the capsaicin+EA group, preoperative capsaicin pretreatment and EA treatment at ST36 acupoint after surgery for 5 days. Capsaicin (TRPV1 agonists, Meilunbio, China) was dissolved in a 10% ethanol, 10% Tween 80, and 0.9% saline mixture. Capsaicin (1 mg/kg) was intraperitoneally injected into rats 3 hours before surgery.

### 2.3. Surgical Procedures

Rats underwent 12 h fasting. Anesthesia was achieved with sodium pentobarbital (50 mg/kg, by intraperitoneal injection). Rats were secured in the supine position, and a laparotomy incision 2 cm long was performed to expose the abdomen after disinfection. Then, after finding the colon, a region 2 cm below the cecum was removed. The incision was closed with a single 6-0 monofilament nylon suture *in situ*. After surgery, the abdomen was sutured in two layers with a 4-0 suture for the muscle fasciae and a 3-0 suture for the skin. Rats were fed a liquid diet for the 48 h after surgery, and then, normal rodent chow and water on day 3. Animals were euthanized by carbon dioxide (CO_2_) on postoperative day 5 to harvest tissues for the detection indexes.

### 2.4. EA Treatment

ST36 is localized on 5 mm below the caput fibulae on the posterolateral side of the knee according to “The Veterinary Acupuncture of China” [13]. Animals in the EA group were fastened to a specially designed holder. After routine disinfection of the skin, stainless-steel needles (Hwato, China), 0.20 × 30 mm in size, were inserted 3 mm deep into the double-sided acupoints. A fine needle placed at ST36 was connected to an electronic acupuncture treatment instrument (Hwato DZ-II, China). The stimulus parameter of amplitude was 1 mA, and the alternate frequency was set to 2/10 Hz. Both EA and sham-EA stimulation was applied for 30 min once daily for a total of 5 d. All EA treatments were conducted at the same time in the morning.

### 2.5. Measuring Defecation Time

Many studies have used the time to the first defecation to measure recovery of postoperative gastrointestinal function [14, 15]. Model rats received a red-carbon-containing liquid (0.5 mL) after surgery. The time from the end of operation to the initial appearance of the first rubescent fecal pellet was recorded.

### 2.6. Detecting Gastric Residue and Gastrointestinal Transit Rate

Gastric residual and gastrointestinal transit rates were done as previously described to assess gastrointestinal function [16, 17]. Prior to the gavage with black semisolid paste (1 mL per rat), animals were fasted for 24 h. Afterward, each rat was sacrificed 30 min later and the stomach and intestine were immediately isolated. The gastric residual rate was calculated by weighing the removed stomach before and after gastric content irrigation. The intestinal propulsion rate was evaluated by the black paste propelling ratio, which was calculated as the percentage of the distance traveled by the maker relative to the total colon length.

### 2.7. Measuring Isolated Gastrointestinal Muscle Motility

Rats were harvested at moribund. Colon tissues were dissected free, cut transversely into 2 cm muscle strips. Each strip was then incubated in the HW200S smooth muscle homeothermic system (Tai Meng Technology Co., Ltd., China) containing 37°C Tyrode's solution (Solarbio, China). All solutions were continuously aerated by a mixed gas (95% O_2_/5% CO_2_). The upper and lower ends of each strip were threaded diagonally and fixed on a tension transducer (FT-102 N, Tai Meng Technology Co., Ltd., China) and the L-shaped hook, respectively. Contractile activities were recorded by a BL-420N laboratory system (Tai Meng Technology Co., Ltd., China) by connecting with the tension transducer.

### 2.8. Mechanical Withdraw Threshold (MWT)

The Von Frey fiber filaments (Stoelting, Wood Dale, IL, USA) were applied to the abdominal incision to assess the MWT. Three different points were selected around abdominal midline incision of each rat for measurement. The measurement was performed 3 times with an interval of 5 min each time. Rats exhibited behaviors including raising their abdomens, scratching, or licking and biting their abdomens, which were considered positive responses. The intensity given at the time of the response was recorded, and the average of 3 measurements was taken.

### 2.9. Spontaneous Nociceptive Behavior Assessment

The rats were placed in plastic cages at room temperature 10 min to adapt to the environment and then observed the behavior for 30 min. Behaviors related to visceral nociception were divided into licking and gnawing on the abdominal or perineal area, body extension, contraction of the flanks, and whole-body contraction. Then, the rat nociception score was calculated using the formula according to the method previously described [18].

### 2.10. Immunohistochemistry

The colon was removed from moribund animals, flushed, and fixed in 4% paraformaldehyde overnight. The 3 *μ*m Paraffin-embedded sections were prepared for immunohistochemistry. After routine dewaxing and gradient hydration, endogenous peroxidase was blocked by H_2_O_2_ (3%). After antigen retrieval by a citrate buffer (pH 6.0), the sections were incubated with anti-TRPV1 (1 : 200, Abcam, UK) and anti-NK1 receptor (1 : 200, Affinity, USA) antibodies at 4°C overnight. The next day, sections were incubated with goat anti-rabbit IgG-HRP-coupled (1 : 1,000, Zhongshan Golden Bridge, China) for 20 min. Finally, the slides were visualized by DAB (Zhongshan Golden Bridge, China) and counterstained with hematoxylin.

### 2.11. Quantitative Real-Time Polymerase Chain Reaction

Total RNA was isolated using TRIzol Reagent (Invitrogen, USA). Subsequently, RNA was reverse transcribed to cDNA using the Reverse Transcription kit (Takara, Japan). Real-time PCR was performed on the ABI 7500 Real-time PCR system (Applied Biosystems, USA). Amplification programs were as follows: 30 s at 95°C and then 40 cycles of amplification (5 s at 95°C and 34 s at 60°C). Each sample was run in triplicate. Target gene expression of each sample was normalized to *β*-actin and calculated by using the 2^−∆∆Ct^ method. The primers used in this study are summarized in Table 1.

### 2.12. Western Blot Analysis

Protein concentration was quantified using a BCA kit (Beyotime, China). Equivalent amounts of protein were separated by 12% SDS-PAGE and then transferred onto PVDF membranes. The membrane was blocked by 5% BSA in TBST for 1 h. The membrane was incubated with primary antibodies against TRPV1 (1 : 1,000, Abcam, UK), NF-*κ*B p65 (1 : 1,000, Proteintech, China), and anti-*β*-actin (1 : 2,000, ImmunoWay, USA) at 4°C overnight. After washing, membranes were incubated with HRP-coupled secondary antibodies (1 : 2,000, Zhongshan Golden Bridge, China). After washing with TBST, immunoblots were visualized with ECL-Plus kit (Beyotime, China). Grey values of immunoreactive bands were normalized to *β*-actin in each sample.

### 2.13. Enzyme-Linked Immunosorbent Assay

Samples collected from the colon tissues were washed with ice-cold PBS and dissected into small fragments. Each sample was weighed and homogenized in PBS at a ratio of 1 : 10 on ice using a glass homogenizer. The freeze-thaw cycle was performed 2 times. The supernatant was obtained after centrifugation at 5,000 × *g* for 5 min. SP level was detected with Rat IL-1*β*, IL-6, TNF-*α*, and SP enzyme-linked immunosorbent assay (ELISA) kits (Jianglai Biological, China). Optical density (OD) value was measured at 450 nm using a microplate reader. The concentration was calculated by plotting a standard curve.

### 2.14. Immunofluorescence

The colon tissue sections were washed 3 times with PBS. Citric acid (pH = 6.0) was used for antigen repair for 5 min. Sections were blocked with 10% normal goat serum for 30 min and incubated with the antibodies against TRPV1 (1 : 100, Abcam, UK) and NF-*κ*B p65 (1 : 200, Proteintech, China) at 4°C overnight. Subsequently, the sections were incubated with the CY3-labeled goat anti-mouse secondary antibody (1 : 800, Proteintech, China) and FITC-labeled goat anti-rabbit secondary antibody (1 : 400, Proteintech, China) for 1 h, and nucleus was stained with DAPI.

### 2.15. Statistical Analysis

The data were analyzed using SPSS 25.0 (SPSS Inc., Chicago, IL, USA). Figures were generated using GraphPad Prism 8 software (GraphPad Software, Inc., San Diego, CA, USA). Data were presented as the mean ± standard deviation (SD). Comparisons between multiple groups were performed using one-way analysis of variance (ANOVA) followed by Tukey's multiple comparison test when the variance was equal. Two-way ANOVA followed by Bonferroni's multiple comparison test was used for analyzing the MWT of skin around abdominal incision and the nociceptive scores. A *t*-test was used for analyzing the differences between model and capsaicin pretreatment groups. A two-sided *P* value less than 0.05 was considered significant.

## 3. Results

### 3.1. EA Treatment Promoted Gastrointestinal Motility in Rats after Colonic Anastomosis

**Figure 1:**
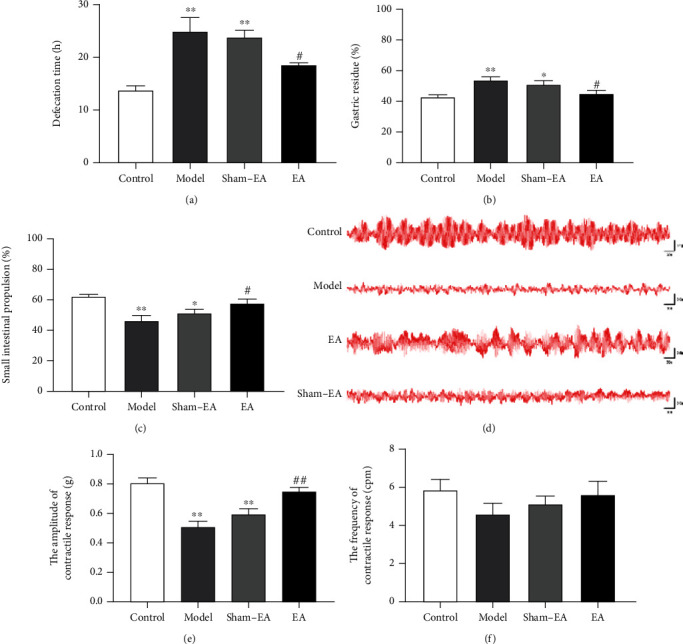
Effects of EA treatment on gastrointestinal motility in rats after colonic anastomosis. (a) Time to the first defecation. All groups were measured by receiving a red-carbon-containing suspension after surgery. (b and c) The gastric residual and intestine propulsion rates were determined *in vivo* using a black semisolid nutrient paste. (d) Contractile activates of isolated muscle strips. (e and f) The amplitude and frequency of the contractile response by isolated muscle strips. (*n* = 8; ^∗^*P* < 0.05 and^∗∗^*P* < 0.01 versus control; ^#^*P* < 0.05 and^##^*P* < 0.01 versus model; one-way ANOVA followed by Tukey post hoc test).

To investigate whether EA treatment affects the gastrointestinal function of normal rats, this study compared the time to the first postoperative defecation of mice in each group. Compared with that in the control group, the time to the first postoperative defecation in the model group was significantly longer (*P* < 0.01, Figure 1(a)). After electroacupuncture treatment, the time to the first defecation was significantly shortened compared with the model (*P* < 0.05, Figure 1(a)). The results showed that the gastric residual rate in the model group was significantly higher than that in the control group (*P* < 0.01, Figure 1(b)). EA treatment significantly reduced gastric remnants (*P* < 0.05, Figure 1(b)). The intestinal propulsion experiment showed that compared with the control group, the distance of the black semisolid nutrient paste in the model rats was shortened (*P* < 0.01, Figure 1(c)). An increased rate of intestinal advancement was observed in EA-treated rats compared with the model (*P* < 0.05, Figure 1(c)). Taken together, these data suggested that EA at ST36 acupoints significantly improved gastrointestinal motility in rats after colonic anastomosis. We studied contractions generated by the smooth muscle using the pressure transducer and homeothermic system to assess the impact of EA therapy. The peristaltic contractile tension of muscle strips from model rats was significantly decreased compared with controls (*P* < 0.01, Figures 1(d) and 1(e)). EA at ST36 increased the contractile tension of isolated muscle strips (*P* < 0.01, Figures 1(e) and 1(e)). As shown in Figure 1(f), the active frequency was not altered by surgery (4.63 ± 1.51 cmp) or EA treatment (5.63 ± 2.00 cmp).

### 3.2. EA Treatment Reduced Postoperative Pain and Inflammation in Rats after Colonic Anastomosis

**Figure 2:**
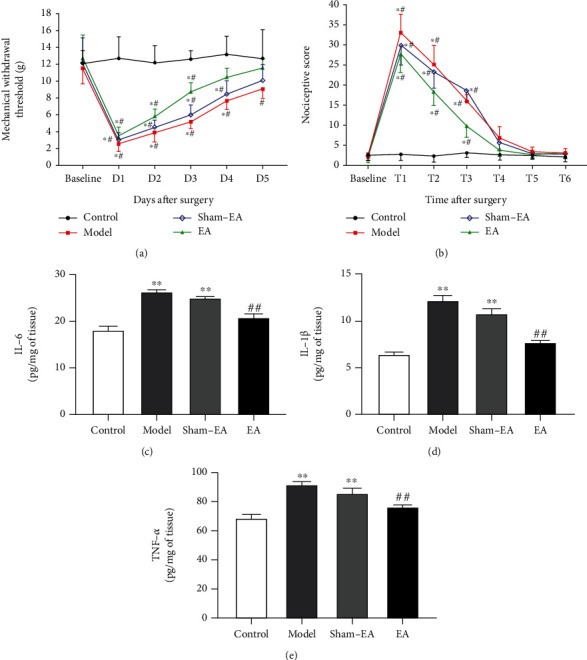
Effects of EA treatment on postoperative pain and inflammation in rats after colonic anastomosis. (a) Changes in the MWT of skin around abdominal incision before and from day 1 to day 5 after surgery in each group in experiment 1. (b) The nociceptive scores of rats before operation (baseline) and at 3 h (T1), 6 h (T2), 12 h (T3), 24 h (T4), 36 h (T5), and 48 h (T6) after surgery in each group in experiment 1. (*n* = 8; ^∗^*P* < 0.05 versus the respective baseline in each group; ^#^*P* < 0.05 versus the corresponding value in the control group; ^Δ^*P* < 0.05 versus the corresponding value in the model group. The MWT and nociceptive scores were analyzed by two-way ANOVA followed by Bonferroni's post hoc test.) (c–e) The expressions of inflammatory cytokines in colon tissues, including IL-6, IL-1*β* and TNF-*α*, were detected by ELISA. (*n* = 8; ^∗∗^*P* < 0.01 versus control; ^##^*P* < 0.01 versus model; one-way ANOVA followed by Tukey's post hoc test).

On days 1 to 3 after colonic anastomosis, the MWT around the abdominal incision in the model group decreased significantly compared with the preoperative basal pain thresholds (*P* < 0.05, Figure 2(a)), whereas there was no statistical difference on days 4 and 5. The sham-EA and EA rats showed a lower MWT around the abdominal incision on days 1 to 4 compared with the corresponding baseline values (*P* < 0.05, Figure 2(a)). Compared with the controls, the MWT around the abdominal incision in the model, sham-EA and EA groups were significantly decreased on days 1 to 3 (*P* < 0.05, Figure 2(a)). There was a significant decrease in the MWT around the abdominal incision in the sham-EA and model groups on days 4 after surgery, while only the model group decreased on days 5 after surgery compared with the control group (*P* < 0.05, Figure 2(a)). The MWT value in the EA group was lower than that in the model group on 3 days after surgery (*P* < 0.05, Figure 2(a)).

The visceral nociceptive scores increased compared with the preoperative basal values at 3 h, 6 h, and 12 h after surgery (*P* < 0.05, Figure 2(b)), while there are no significant changes at 24 h, 36 h, and 48 h after surgery. There was a significant increase in the visceral nociceptive scores in the model, sham-EA, and EA groups at 3 h, 6 h, and 12 h after surgery (*P* < 0.05, Figure 2(b)). Compared with the models, EA treatment significantly reduced the visceral nociceptive behavior in rats at 3 h, 6 h, and 12 h after surgery (*P* < 0.05, Figure 2(b)).

To investigate the effect of electroacupuncture treatment on the inflammatory profile of rats after surgery, we examined the expression levels of IL-6, IL-1*β*, and TNF-*α* of rats in each group 5 days after surgery. Compared with controls, the expressions of IL-6, IL-1*β*, and TNF-*α* were significantly increased in models and sham-EA rats (*P* < 0.01, Figures 2(a)–2(c)). The expression levels of IL-6 and IL-1*β* were significantly lower in the EA rats compared with the model group (*P* < 0.01, Figures 2(a) and 2(b)), while the expression level of TNF-*α* was also significantly decreased in the EA group (*P* < 0.01, Figure 2(c)).

### 3.3. EA Treatment Reduced TRPV1 Expression in the Colon Tissue of Rats after Colonic Anastomosis

**Figure 3:**
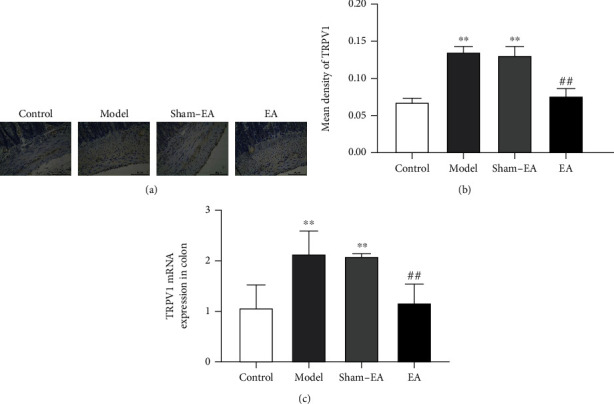
Effects of EA treatment on the expression of TRPV1 in rats after colonic anastomosis. (a) Immunohistochemical staining for TRPV1 in colon tissues (×400, scale bar = 50 *μ*m). (b) The mean density of TRPV1 positive staining in colon tissues. (c) The expression of TRPV1 mRNA in colon tissues of rats in each group was detected by quantitative real-time PCR. (*n* = 8; ^∗∗^*P* < 0.01 versus control; ^##^*P* < 0.01 versus model; one-way ANOVA followed by Tukey's post hoc test).

To explore the potential mechanism of EA treatment at ST36 acupoint in rats after colonic anastomosis, we detected the expression level of TRPV1. Immunohistochemistry results indicated that compared with the control group, TRPV1 levels were increased in the colon of the model and sham-EA rats (*P* < 0.01, Figures 3(a) and 3(b)). The EA group showed reduced TRPV1 expression (*P* < 0.01, Figures 3(a) and 3(b)), almost to normal levels. Meanwhile, PCR analysis of colon tissue from model and sham-EA rats showed that TRPV1 mRNA expression was obviously increased compared with controls (*P* < 0.01, Figure 3(c)). However, TRPV1 mRNA levels were significantly decreased in the EA rats compared with the models (*P* < 0.01, Figure 3(c)).

### 3.4. EA Treatment Reduced SP/NK1 Receptor and NF-*κ*B Protein Expression in the Colon Tissue of Rats after Colonic Anastomosis

**Figure 4:**
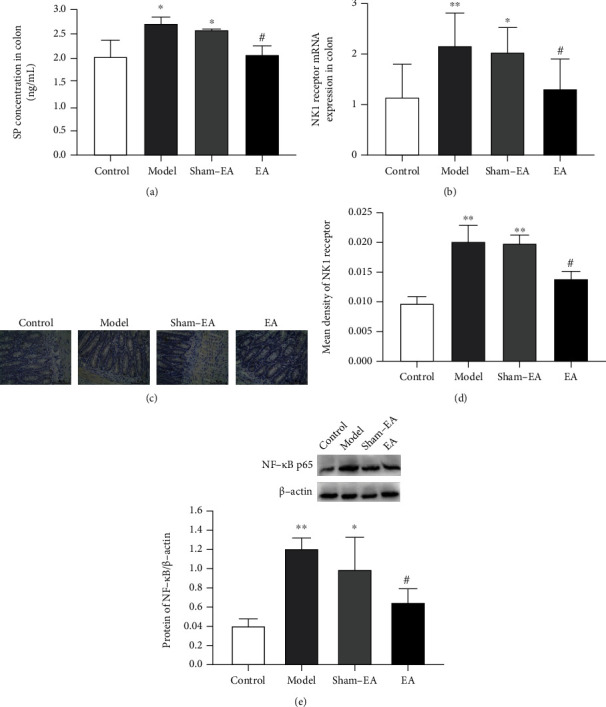
Effects of EA treatment on the SP/NK1 receptor binding and NF-*κ*B expression in rats after colonic anastomosis. (a) SP levels in the colon tissues were calculated using ELISA kits. (b) The expression of NK1 receptor mRNA in colon tissues of rats in each group was detected by quantitative real-time PCR. (c) Immunohistochemical staining for NK1 receptor in intestinal tissues (×400, scale bar = 50 *μ*m). (d) The mean density of NK1 receptor-positive staining in colon tissues. (e) NF-*κ*B (p65) protein expression in colon tissues of rats in each group was detected by western blot. (*n* = 8; ^∗^*P* < 0.05 and^∗∗^*P* < 0.01 versus control; ^#^*P* < 0.05 versus model; one-way ANOVA followed by Tukey's post hoc test).

ELISA results suggested that the model group had significantly elevated SP levels in the colon tissue compared with the controls (*P* < 0.05, Figure 4(a)). However, the increased SP values declined distinctly after EA treatment (*P* < 0.05, Figure 4(a)). Our PCR results revealed an upregulated NK1 receptor mRNA expression in the model and sham-EA groups (*P* < 0.01 and *P* < 0.05, Figure 4(b)), and EA stimulation at ST36 acupoint reversed the upregulation of NK1 receptor mRNA expression in rats after colonic anastomosis (*P* < 0.05, Figure 4(b)). Immunohistochemistry revealed that NK1 receptor expression in the colon was markedly increased in the model and sham-EA groups (*P* < 0.01 and *P* < 0.01, Figures 4(c) and 4(d)). After EA treatment, NK1 receptor expression was significantly decreased compared with the models (*P* < 0.05, Figures 4(c) and 4(d)). Our experiment also detected the expression of NF-*κ*B (p65) protein in the colon tissue of rats in each group; the results showed a significantly increased expression in rats after colonic anastomosis (*P* < 0.01, Figure 4(e)). The expression of NF-*κ*B (p65) protein was significantly higher in sham-EA rats compared with the controls (*P* < 0.05, Figure 4(e)). In contrast to the model group, the protein expression of NF-*κ*B (p65) was reduced in the EA group (*P* < 0.05, Figure 4(e)).

### 3.5. TRPV1 and NF-*κ*B Were Colocalized in Colon Tissue

**Figure 5:**
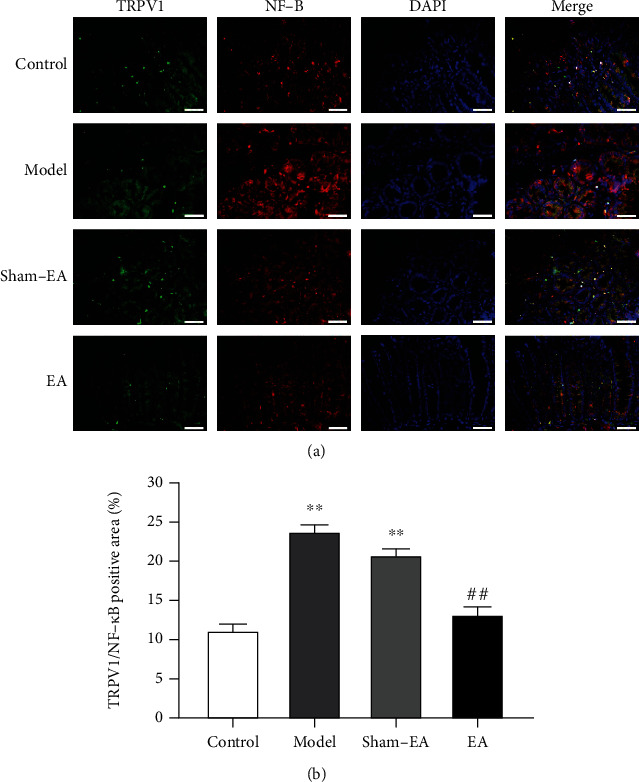
TRPV1 and NF-*κ*B were colocalized in colon tissues. (a) Immunofluorescence images of colon tissue sections of rats in each group. TRPV1-positive immunoreaction is shown in green, NF-*κ*B-positive immunoreaction is shown in red, and nuclear nucleus staining with DAPI is shown in blue. Merge images show a superimposed image of triple markers. Magnification: ×400; scale bar = 50 *μ*m. (b) Quantitative expressions of TRPV1 and NF-*κ*B in tissue sections of different groups and statistical analysis. (*n* = 8; ^∗∗^*P* < 0.01 versus control; ^##^*P* < 0.01 versus model; one-way ANOVA followed by Tukey's post hoc test).

As shown in Figure 5, we localized TRPV1 and NF-*κ*B in the colon tissue of each group of rats by immunofluorescence technique. The results showed that TRPV1 and NF-*κ*B were colocalized in the colon, primarily distributed in the mucosa and submucosa. The expressions of TRPV1 and NF-*κ*B were significantly increased in the model and sham-EA rats compared with the controls (*P* < 0.01, Figures 5(a) and 5(b)). In contrast, the expressions of TRPV1 and NF-*κ*B were reduced in postoperative rats after EA treatment (*P* < 0.01, Figures 5(a) and 5(b)).

### 3.6. EA Treatment Inhibited NF-*κ*B Expression *via* TRPV1

**Figure 6:**
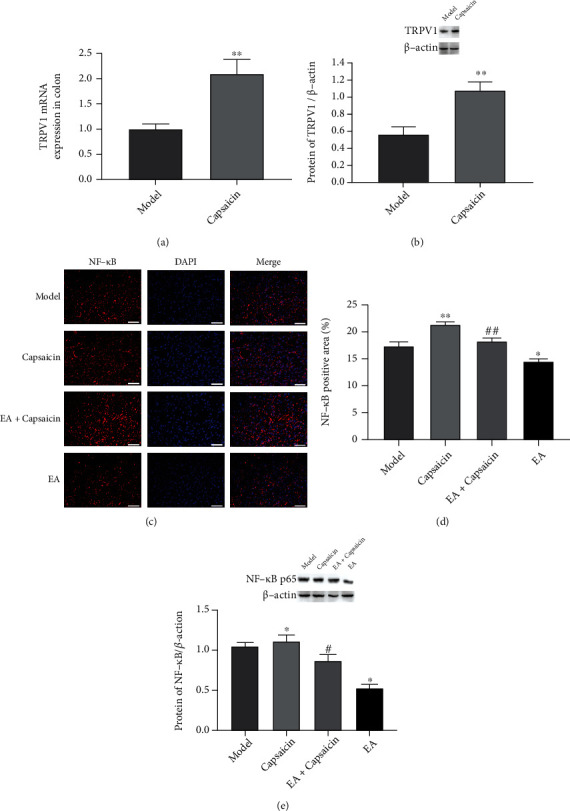
Effects of EA TRPV1 agonist on the expression of NF-*κ*B in rats after colonic anastomosis. (a and b) Rats were pretreated with capsaicin (TRPV1 agonist) before colon anastomosis. The expressions of TPRV1 mRNA and protein in colon tissues were detected in each group. (*n* = 8; ^∗∗^*P* < 0.01 versus model; independent sample *t*-test.) (c) Immunofluorescence images of colon tissue sections of rats in each group. NF-*κ*B-positive immunoreaction showed red, and DAPI staining showed blue nuclei. Magnification: ×400; scale bar = 50 *μ*m. (d) Quantitative expression of NF-*κ*B in tissue sections of different groups and statistical analysis. (e) NF-*κ*B (p65) protein expression in colon tissues of rats in each group was detected by western blot assay. (*n* = 8; ^∗^*P* < 0.05 and^∗∗^*P* < 0.01 versus model; ^#^*P* < 0.05 and^##^*P* < 0.01 versus EA; one-way ANOVA followed by Tukey's post hoc test).

In experiment 2, the therapeutic effect of EA on rats after colonic anastomosis was further evaluated using capsaicin (TRPV1 agonist). The results showed that capsaicin as a specific agonist significantly enhanced the TRPV1 mRNA and protein expression in rats after colonic anastomosis (*P* < 0.01, Figure 6(a); *P* < 0.01, Figure 6(b)). In addition, immunofluorescence results showed a significant increase of NF-*κ*B expression was found in the colon tissues of postoperative rats pretreated with capsaicin (*P* < 0.01, Figures 6(c) and 6(d)). EA treatment reduced the expression of NF-*κ*B in postoperative rats pretreatment with capsaicin (*P* < 0.01, Figures 6(c) and 6(d)). Compared with the model group, there was a significant increase expression of NF-*κ*B (p65) protein in the capsaicin group (*P* < 0.05, Figure 6(e)). However, pretreatment with capsaicin increased the expression of NF-*κ*B (p65) protein in postoperative rats compared with the electroacupuncture group (*P* < 0.05, Figure 6(e)).

### 3.7. Involvement of TRPV1 in the Treatment of Postoperative Inflammation and Pain in Rats by EA

**Figure 7:**
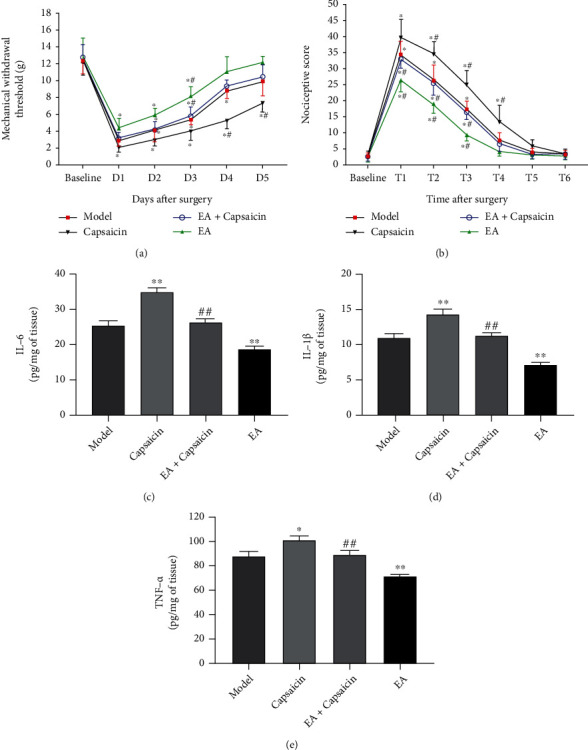
Effects of EA TRPV1 agonist on postoperative pain and inflammation in rats after colonic anastomosis. (a) Changes in the MWT of skin around abdominal incision before and from day 1 to day 5 after surgery in each group. (b) The nociceptive scores of rats before operation (Baseline) and at 3 h (T1), 6 h (T2), 12 h (T3), 24 h (T4), 36 h (T5), and 48 h (T6) after surgery in each group. (*n* = 8; ^∗^*P* < 0.05 versus the respective baseline in each group; ^#^*P* < 0.05 versus the corresponding value in the model group; ^Δ^*P* < 0.05 versus the corresponding value in the EA group. The MWT and nociceptive scores were analyzed by two-way ANOVA followed by Bonferroni's post hoc test.) (c–f) The expressions of inflammatory cytokines in colon tissues, including IL-6, IL-1*β*, and TNF-*α*, were detected by ELISA. (^∗^*P* < 0.05 and^∗∗^*P* < 0.01 versus control; ^##^*P* < 0.01 versus EA; one-way ANOVA followed by Tukey's post hoc test).

In terms of postoperative pain, it was found that the MWT around the abdominal incision in the model and EA+capsaicin groups from days 1 to 4 decreased significantly compared with the baselines (*P* < 0.05, Figure 7(a)). The MWT values were decreased in the capsaicin group on days 1 to 5 and in the EA group on days 1 to 3 compared with the corresponding basal MWT values (*P* < 0.05, Figure 7(a)). Compared with the models, the MWT was significantly decreased in the capsaicin group on days 4 and 5 (*P* < 0.05, Figure 7(a)), whereas it was markedly increased in the EA group on day 3 (*P* < 0.05, Figure 7(a)). On day 3 after colonic anastomosis, the MWT in the EA+capsaicin group was obviously reduced compared with the EA group (*P* < 0.05, Figure 7(a)).

The behavioral results revealed that the nociceptive behavior at 3 h, 6 h, and 12 h after surgery markedly increased in each group compared with the baseline (*P* < 0.05, Figure 7(b)). However, the visceral nociceptive score was only higher than the basal value in the capsaicin group at 24 h after surgery (*P* < 0.05, Figure 7(b)). In comparison with the models, the visceral nociceptive score was significantly elevated in the capsaicin group at 6 h, 12 h, and 24 h postoperatively (*P* < 0.05, Figure 7(b)), while it was decreased in the EA group at 3 h, 6 h, and 12 h after surgery (*P* < 0.05, Figure 7(b)). In addition, spontaneous behavior was significantly increased in the EA+capsaicin group compared with the EA group at 3 h, 6 h and 12 h after surgery (*P* < 0.05, Figure 7(b)).

We detected the expression levels of IL-6, IL-1*β*, and TNF-*α* to explore the effect of TRPV1 agonists on the inflammation of the postoperative rats in each group. The results showed that the expression levels of IL-6, IL-1*β*, and TNF-*α* were significantly higher in the capsaicin group (*P* < 0.01, *P* < 0.01, and *P* < 0.05; Figures 7(c)–7(e)) and lower in the EA group compared with the models (*P* < 0.01, *P* < 0.01, and *P* < 0.01; Figures 7(c)–7(e)). Compared to the EA group, the IL-6, IL-1*β*, and TNF-*α* levels of the EA+capsaicin group increased notably (*P* < 0.01, *P* < 0.01, and *P* < 0.01; Figures 7(c)–7(e)).

## 4. Discussion

Postoperative gastrointestinal function is easily affected by nerves, immunity, and inflammation, thus affecting postoperative gastrointestinal motility. Different anesthesia methods, surgical methods, stimulation of the gastrointestinal tract by surgery, and the patient's constitution can all cause postoperative gastrointestinal dysfunction [19]. Previous studies have shown that the suppression of gastrointestinal motility is mainly manifested by the first postoperative defecation time and the decreased gastrointestinal motility [20, 21]. Previous studies have shown that about 10% to 50% of patients who undergo surgery due to different conditions may develop persistent pain syndrome after surgery [22]. Monocytes and neutrophils of the macrophage population enter the intestinal muscle within 24 h after intestinal manipulation, further recruiting more macrophages that promote the release of inflammatory cytokines, thereby triggering intestinal inflammation through the release of IL-6 and TNF-*α*, which are the main causes of gastrointestinal motility disorders associated with postoperative ileus [23]. Consistent with the previous findings, our experimental results successfully demonstrate a suppression of gastrointestinal motility in rats after colonic anastomosis, accompanied by postoperative pain and inflammatory response.

Previous studies have shown that acupuncture treatment can not only improve the digestion, secretion, and absorption capacity of the gastrointestinal tract but also effectively relieve gastrointestinal spasm, postoperative intestinal obstruction, postoperative visceral pain, nausea, vomiting, and other functional gastrointestinal diseases [24–26]. EA was effective in improving the MWT on both the contralateral and ipsilateral sides of the rats after knee surgery, reducing cumulative pain scores and decreasing mechanical hypersensitivity of knee [27]. EA at ST36 acupoint reduced serum IL-6, IL-1*β*, and TNF-*α* levels in mice with acute colitis induced by dextran sulfate sodium [28]. In this study, we observed that EA treatment shortened the first postoperative defecation time and accelerated gastrointestinal motility in a rat model after colonic anastomosis. In addition, the results demonstrated a reduced expression of IL-6, IL-1*β*, and TNF-*α*; an increased incision MWT; and a decreased spontaneous nociceptive behavior in rats after EA treatment. These suggest that EA treatment may promote the recovery of gastrointestinal motility in rats after colonic anastomosis, inhibit postoperative inflammatory response, and relieve postoperative pain, but the mechanism is not clear.

In the present study, we found that TRPV1 was expressed in both mucosa and submucosa of colon tissue by immunohistochemistry and immunofluorescence localization. Previous studies have shown that the activation of TRPV1 enables the influx of extracellular Ca^2+^ and Na^+^, causing the release of neuropeptides, thereby activating some pathophysiological processes, such as pain signal transduction, inflammatory responses, neuroplasticity regulation, and systemic immunity [29, 30]. A study has shown that EA at ST36 acupoint can regulate the expression and activation of TRPV1 and TRPV4 involved in spinal cord central sensitization [31]. At the same time, a study has found that the mechanism of EA at ST36 acupoint to relieve cancer pain is related to the expression of TRPV1 [32]. However, the mechanism of whether EA at ST36 acupoint can improve postoperative gastrointestinal motility, postoperative pain, and inflammation caused by colon surgery *via* TRPV1 is still unclear. In this study, we found that EA treatment could reduce the increased expression of TRPV1, IL-6, IL-1*β*, TNF-*α*, and the abdominal incision skin MWT and decrease the spontaneous nociceptive scores in rats caused by colon anastomosis. Therefore, these suggest that EA may improve postoperative gastrointestinal motility and relieve postoperative pain and inflammatory response *via* mediating TRPV1.

In addition, the present study suggests that EA may decrease the increased expression levels of SP and NK1 receptor in a rat model after colonic anastomosis. SP plays an important role in gastrointestinal motility, secretion, pain, and immune function as a key neurotransmitter of injurious signals [33]. It preferentially activates NK1 receptors and plays a central role in the regulation of interstitial cell of Cajal (ICC) potentiation and gastrointestinal smooth muscle contraction [34, 35]. Previous studies have shown that afferent nerve endings of TRPV1 can release SP and SP can also activate TRPV1 by phosphorylation, increasing the possibility of channel gating [36]. SP recruited leukocytes to the peripheral terminals of nociceptors and releases neuroactive mediators that contribute to neuropathic pain [34, 37]. Therefore, EA appears to improve postoperative gastrointestinal motility and reduce postoperative inflammatory and pain responses *via* regulating TRPV1-mediated SP/NK1 receptor binding.

In the present study, our immunofluorescence staining data showed substantial colocalization of TRPV1 and NF-*κ*B in colon tissue. Previous data suggest that EA exerts anti-inflammatory effects in a rat tissue chamber model by inhibiting nuclear translocation of NF-*κ*B p65 and increasing the expression of NF-*κ*B I*κ*B*α* [38]. It has also been shown that the expression of IL-1*β*, IL-6, and TNF-*α* can be downregulated by inhibiting gene transcription and the expression of NF-*κ*B, SP, and NK1 receptors, suggesting that inhibition of NF-*κ*B and SP/NK1 receptor binding may attenuate the inflammatory response [39]. Nevertheless, the possible link between TRPV1 and NF-*κ*B in the EA treatment at ST36 acupoint of gastrointestinal motility and inflammatory pain after colonic anastomosis remains to be verified.

To further investigate the association of TRPV1 and NF-*κ*B in EA treatment in rats after colonic anastomosis, rats were pretreated with capsaicin, a potent TRPV1 agonist, prior to colonic anastomosis [40]. EA stimulation exerted a protective effect on the colonic mucosa of rats with ulcerative colitis by inhibiting the NF-*κ*B signaling pathway and reducing the release of inflammatory cytokines [41]. Sodium hydrosulfide regulated gastric acid secretion by mediating TRPV1 activation in sensory nerve endings and subsequent release of SP in an NF-*κ*B-dependent manner [42]. In the present study, preoperative capsaicin pretreatment upregulated NF-*κ*B p65 protein and SP expression in rat colon tissue after colonic anastomosis, suggesting that TRPV1 may have a regulatory effect on NF-*κ*B and SP. Our results show a decreased postoperative incision MWT, an increased spontaneous injurious behavior, and an upregulated expression of the inflammatory cytokines IL-6, IL-1*β*, and TNF-*α* in rats undergoing colonic anastomosis after application of capsaicin pretreatment. However4, these findings could be reversed by EA at ST36 acupoint treatment. Therefore, EA may improve gastrointestinal motility, pain, and inflammation in rats after colonic anastomosis by affecting TRPV1, SP/NK1 receptor binding, and NF-*κ*B expression.

We investigated the effects and mechanisms of EA at ST36 acupoint on the peristaltic function of the gastrointestinal tract in rats after colonic anastomosis by in vivo and vitro experiments. However, we did not examine other types of signaling pathway-related factors, such as TRPA1 signaling pathway, macrophages, glial cells, chemokines, and many other cytokines.

In conclusion, our findings suggest that electroacupuncture at ST36 acupoint can improve gastrointestinal motility in rats after colonic anastomosis and reduce postoperative intestinal inflammation and pain. This mechanism may be mediated by inhibiting the activation of NF-*κ*B and SP/NK1 receptor signaling pathways by inhibiting TRPV1 expression. At the same time, our research provides a new molecular mechanism and theoretical basis for acupuncture to improve gastrointestinal function and also provides a new treatment idea for clinically promoting the recovery of gastrointestinal function after colon surgery.

## Figures and Tables

**Table 1:** The primers used in this study.

Target gene	Primer name	Sequence (5′-3′)
*β*-Actin	*β*-Actin forward	CCCTAAGGCCAACCGTGAAAAG
*β*-Actin	*β*-Actin reverse	ACCCTCATAGATGGGCACAGT
TRPV1	TRPV1 forward	ACTGTACTTCAGCCAACGCA
TRPV1	TRPV1 reverse	TCTGCTGGAATCCTCGGGTA
NK1 receptor	NK1 receptor forward	GCAATAGACATGGCTGGATTTG
NK1 receptor	NK1 receptor reverse	GACAAGTTCTCGAAGGAGACTG

## Data Availability

All data were included in the manuscript.

## References

[1] Chon T. Y., Lee M. C. (2013). Acupuncture. *Mayo Clinic Proceedings*.

[2] Takahashi T. (2013). Effect and mechanism of acupuncture on gastrointestinal diseases. *International Review of Neurobiology*.

[3] Zhao Y. X., Cui C. X., Gao J. H. (2021). Electroacupuncture ameliorates corticotrophin-releasing factor-induced jejunal dysmotility in a rat model of stress. *Acupuncture in Medicine*.

[4] Tang L., Zeng Y., Li L. (2020). Electroacupuncture upregulated ghrelin in rats with functional dyspepsia via AMPK/TSC2/Rheb-mediated mTOR inhibition. *Digestive Diseases and Sciences*.

[5] Kargbo R. B. (2019). TRPV1 modulators for the treatment of pain and inflammation. *ACS Medicinal Chemistry Letters*.

[6] Pei L., Chen H., Guo J. (2018). Effect of acupuncture and its influence on visceral hypersensitivity in IBS-D patients: study protocol for a randomized controlled trial. *Medicine (Baltimore)*.

[7] Shi H. L., Liu C. H., Ding L. L. (2015). Alterations in serotonin, transient receptor potential channels and protease-activated receptors in rats with irritable bowel syndrome attenuated by Shugan decoction. *World Journal of Gastroenterology*.

[8] Yu Z., Zhang N., Lu C.-X. (2016). Electroacupuncture at ST25 inhibits jejunal motility: role of sympathetic pathways and TRPV1. *World Journal of Gastroenterology*.

[9] Yang J., Hsieh C. L., Lin Y. W. (2017). Role of transient receptor potential vanilloid 1 in electroacupuncture analgesia on chronic inflammatory pain in mice. *BioMed Research International*.

[10] Lin T. H., Pajarinen J., Lu L. (2017). NF-*κ*B as a therapeutic target in inflammatory-associated bone diseases. *Advances in Protein Chemistry and Structural Biology*.

[11] Zhang K., Zhao X., Yang T. (2021). Initiation of acupoint molecular mechanisms for manual acupuncture analgesia-nuclear factor *κ*B signaling pathway. *Chinese Journal of Integrative Medicine*.

[12] Li N., Guo Y., Gong Y. (2021). The anti-inflammatory actions and mechanisms of acupuncture from acupoint to target organs via neuro-immune regulation. *Journal of Inflammation Research*.

[13] Ma F. Q., Sun C. J., Wei J. J., Wang Y. D., Shen J. C., Chang J. J. (2020). Electro-acupuncture regulates glucose metabolism in chronic stress model rats. *Scientific Reports*.

[14] Fraser G. L., Venkova K., Hoveyda H. R., Thomas H., Greenwood-Van M. B. (2009). Effect of the ghrelin receptor agonist TZP-101 on colonic transit in a rat model of postoperative ileus. *European Journal of Pharmacology*.

[15] Liang C., Wang K. Y., Gong M. R., Li Q., Yu Z., Xu B. (2018). Electro-acupuncture at ST37 and ST25 induce different effects on colonic motility via the enteric nervous system by affecting excitatory and inhibitory neurons. *Neurogastroenterology and Motility*.

[16] Cai G. X., Liu B. Y., Yi J., Chen X. M., Liu F. L. (2011). Simotang enhances gastrointestinal motility, motilin and cholecystokinin expression in chronically stressed mice. *World Journal of Gastroenterology*.

[17] Liang Q., Yan Y., Mao L. (2018). Evaluation of a modified rat model for functional dyspepsia. *Saudi Journal of Gastroenterology*.

[18] Huang J., Liu L., Zhou Y., Yu J., Deng J. (2006). Suppressive effects of intrathecal application of diazepam on visceral pain and hyperalgesia induced by intracolonic instillation of formalin. *International Journal of Biomedical Sciences*.

[19] Zheng Y., Qin Z., Tsoi B., Shen J., Zhang Z. J. (2020). Electroacupuncture on trigeminal nerve-innervated acupoints ameliorates poststroke cognitive impairment in rats with middle cerebral artery occlusion: involvement of neuroprotection and synaptic plasticity. *Neural Plasticity*.

[20] Chen K. B., Huang Y., Jin X. L., Chen G. F. (2019). Electroacupuncture or transcutaneous electroacupuncture for postoperative ileus after abdominal surgery: a systematic review and meta-analysis. *International Journal of Surgery*.

[21] Guo Y., Kong X., Cao Q. (2021). Efficacy and safety of acupuncture in postoperative ileus after gynecological surgery: a protocol for system review and meta-analysis of randomized controlled trials. *Medicine (Baltimore)*.

[22] Kehlet H. (2018). Postoperative pain, analgesia, and recovery-bedfellows that cannot be ignored. *Pain*.

[23] Yang N. N., Yang J. W., Ye Y. (2021). Electroacupuncture ameliorates intestinal inflammation by activating *α*7nAChR-mediated JAK2/STAT3 signaling pathway in postoperative ileus. *Theranostics*.

[24] Li Y., Yang M., Wu F. (2019). Mechanism of electroacupuncture on inflammatory pain: neural-immune-endocrine interactions. *Journal of Traditional Chinese Medicine*.

[25] Lan L., Zeng F., Liu G. J. (2014). Acupuncture for functional dyspepsia. *Cochrane Database of Systematic Reviews*.

[26] Mao X., Guo S., Ni W. (2020). Electroacupuncture for the treatment of functional dyspepsia: a systematic review and meta-analysis. *Medicine (Baltimore)*.

[27] Huang H., Song X., Wang J. (2021). Opposing and operated side electroacupuncture generates similar analgesic effects on pain after knee surgery. *Evidence-based Complementary and Alternative Medicine*.

[28] Song S., An J., Li Y., Liu S. (2019). Electroacupuncture at ST-36 ameliorates DSS-induced acute colitis via regulating macrophage polarization induced by suppressing NLRP3/IL-1*β* and promoting Nrf2/HO-1. *Molecular Immunology*.

[29] Moriello A. S., De Petrocellis L. (2016). Assay of TRPV1 receptor signaling. *Methods in Molecular Biology*.

[30] Yang F., Zheng J. (2017). Understand spiciness: mechanism of TRPV1 channel activation by capsaicin. *Protein & Cell*.

[31] Lin J. G., Hsieh C. L., Lin Y. W. (2015). Analgesic effect of electroacupuncture in a mouse fibromyalgia model: roles of TRPV1, TRPV4, and pERK. *PLoS One*.

[32] Zhang Z., Wang C., Gu G. (2012). The effects of electroacupuncture at the ST36 (Zusanli) acupoint on cancer pain and transient receptor potential vanilloid subfamily 1 expression in Walker 256 tumor-bearing rats. *Anesthesia and Analgesia*.

[33] Shang J. J., Yuan J. Y., Xu H., Tang R. Z., Dong Y. B., Xie J. Q. (2013). Shugan-decoction relieves visceral hyperalgesia and reduces TRPV1 and SP colon expression. *World Journal of Gastroenterology*.

[34] Sousa-Valente J., Brain S. D. (2018). A historical perspective on the role of sensory nerves in neurogenic inflammation. *Seminars in Immunopathology*.

[35] Suvas S. (2017). Role of substance P neuropeptide in inflammation, wound healing, and tissue homeostasis. *Journal of Immunology*.

[36] Allais L., De Smet R., Verschuere S., Talavera K., Cuvelier C. A., Maes T. (2016). Transient receptor potential channels in intestinal inflammation: what is the impact of cigarette smoking?. *Pathobiology*.

[37] Chen Y., Mu J., Zhu M., Mukherjee A., Zhang H. (2020). Transient receptor potential channels and inflammatory bowel disease. *Frontiers in Immunology*.

[38] Liu F., Fang J., Shao X., Liang Y., Wu Y., Jin Y. (2014). Electroacupuncture exerts an anti-inflammatory effect in a rat tissue chamber model of inflammation via suppression of NF-*κ*B activation. *Acupuncture in Medicine*.

[39] Yu Y., Zhu W., Liang Q., Liu J., Yang X., Sun G. (2018). Tropisetron attenuates lipopolysaccharide induced neuroinflammation by inhibiting NF-*κ*B and SP/NK1R signaling pathway. *Journal of Neuroimmunology*.

[40] Bujak J. K., Kosmala D., Szopa I. M., Majchrzak K., Bednarczyk P. (2019). Inflammation, cancer and immunity-implication of TRPV1 channel. *Frontiers in Oncology*.

[41] Qiao C. X., Zhao G., Zhang L. Z. (2020). Intervention mechanism of electroacupuncture in rats with ulcerative colitis: an analysis based on the Toll-like receptor 4/myeloid differentiation factor 88/nuclear factor-kappa B signaling pathway. *Zhen Ci Yan Jiu*.

[42] Sun H. Z., Gong X. Y., Wu L. (2018). Hydrogen sulfide modulates gastric acid secretion in rats via involvement of substance P and nuclear factor-*κ*B signaling. *Journal of Physiology and Pharmacology*.

